# Interplay Between Infectious Diseases and the Endocrine System: An Overview and Clinical Insights

**DOI:** 10.1002/edm2.70259

**Published:** 2026-07-02

**Authors:** Francesco Capoccia, Valentina D'Onghia, Valentina Semeraro, Esposito Susanna, Maria Elisabeth Street

**Affiliations:** ^1^ Department of Medicine and Surgery University of Parma Parma Italy; ^2^ Pediatric Clinic University Hospital of Parma Parma Italy

**Keywords:** adrenalitis, diabetes mellitus type 1, endocrine dysfunction, hypocortisolism, hypogonadism, hypopituitarism, hypothyroidism, infections, thyroiditis, Waterhouse Friderichsen syndrome

## Abstract

**Introduction:**

Infectious diseases can affect the endocrine system through multiple mechanisms, including direct cytopathic effects, immune dysregulation, inflammatory pathways, and in some cases the secretion of hormone‐like substances by the pathogens themselves. While viral and bacterial infections represent the most frequent triggers, fungal and parasitic agents can also contribute, though less commonly.

**Methods:**

This narrative review, based on a search in PubMed and Google Scholar, examined both paediatric and adult studies, given the limited evidence available in children. The analysis focused on the pituitary, thyroid, adrenal glands, gonads and endocrine pancreas, summarising current knowledge on the acute and long‐term endocrine consequences of infections.

**Results:**

Reported outcomes range from type 1 diabetes mellitus and adrenal insufficiency to hypopituitarism, thyroiditis and hypogonadism. These complications may appear during the acute phase or emerge later as delayed sequelae, with significant implications for prognosis, quality of life and overall morbidity. The review highlights the need for increased clinical awareness, timely recognition of infection‐related endocrine dysfunctions, and structured follow‐up to prevent long‐lasting complications.

**Conclusion:**

Further translational and clinical studies are warranted to clarify underlying mechanisms, identify early biomarkers and develop evidence‐based diagnostic and therapeutic strategies, ultimately improving patient care and outcomes in both paediatric and adult populations. In the future, through genomic and transcriptomic techniques, the identification of the dysregulated cellular pathways could help clarify the real ‘cause‐and‐effect’ relationship between infections and cellular dysfunction.

## Graphical abstract

Infectious diseases can disrupt endocrine homeostasis through direct cytopathic injury, autoimmune dysregulation, or pathogen‐derived hormone‐like effects. Viruses and bacteria are the most frequent agents, whereas fungi and parasites contribute less commonly, even remaining clinically relevant, especially in the immunocompromised population. All glands can be affected, resulting in acute or late‐onset presentations of hypopituitarism, SIADH, diabetes insipidus, hypothyroidism, adrenal insufficiency, hypogonadism, or type 1 diabetes. These findings support the importance of early clinical recognition and of a structured endocrinological follow‐up in addition to the need of integrating emerging research tools to clarify the real cause‐effect relationship between infections and endocrine damage.

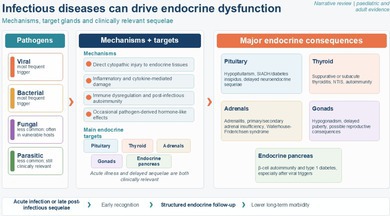

## 1 Introduction

Infectious diseases exert profound and often underestimated effects on the endocrine system. Endocrine disruption may occur during both acute and chronic infections through a range of mechanisms, including direct cytopathic effects on endocrine tissues, inflammatory and cytokine‐mediated damage, dysregulation of the immune system with subsequent autoimmunity, or even pathogen‐driven secretion of hormone‐like substances [1]. Such interactions highlight the complex and bidirectional relationship between infections and endocrine physiology, where not only do infections alter hormonal balance, but endocrine dysfunction itself can influence immune competence and susceptibility to pathogens.

Viral and bacterial pathogens represent the most frequent infectious that can cause endocrine abnormalities [2]. Classic examples include viral infections as triggers for type 1 diabetes mellitus [3] or bacterial meningitis leading to pituitary dysfunction [4]. Nevertheless, fungal and parasitic infections—though less common—can also have clinically significant consequences, particularly in immunocompromised individuals [5]. Reported endocrine outcomes are wide‐ranging, from hypothyroidism, adrenal insufficiency and hypogonadism to conditions such as hypopituitarism, thyroiditis, or Waterhouse–Friderichsen syndrome. Importantly, these abnormalities may present acutely, sometimes with life‐threatening consequences, or emerge years later as delayed sequelae, adding to the long‐term burden of morbidity.

Age represents another critical determinant. Much of the existing evidence derives from adult populations, where the prevalence and spectrum of endocrine sequelae are better characterised. Paediatric data remain limited, fragmented and often contradictory. For example, while children may exhibit a lower vulnerability to certain post‐infectious complications, such as pituitary insufficiency after meningitis [6] they remain highly susceptible to others, such as acute suppurative thyroiditis [7]. This underscores the importance of age‐specific considerations in both research and clinical care.

Despite growing recognition of infection‐related endocrine sequelae, clinical awareness remains insufficient, leading to underdiagnosis and delays in treatment. Structured endocrine follow‐up after severe infections is rarely standardised, and evidence‐based guidelines are lacking [8]. Moreover, current literature is constrained by methodological heterogeneity, limited follow‐up durations, and the absence of early biomarkers to identify individuals at highest risk.

The aim of this narrative review is to provide a synthesis of available evidence on the interplay between infectious diseases and endocrine dysfunction. By collating current knowledge, this review seeks to improve clinical awareness for earlier recognition and intervention.

## 2 Materials and Methods

The literature search was performed in PubMed and Google Scholar, with complementary searches in Scopus and Web of Science to identify additional relevant publications and to review reference lists of selected articles. The search covered studies published from January 1980 to September 2025, and the last search was conducted in September 2025.

The search strategy was intentionally broad to capture the heterogeneity of available evidence across endocrine systems and infectious etiologies. In PubMed, Medical Subject Headings (MeSH) were used when available. Articles were retrieved when the major topic corresponded to predefined endocrine disorders, including hypopituitarism, pituitary insufficiency/dysfunction, thyroid dysfunction, thyroiditis, hypocortisolism, adrenal insufficiency/dysfunction, Addison disease, Waterhouse–Friderichsen syndrome, Cushing syndrome, hypogonadism and type 1 diabetes mellitus. These terms were combined using the Boolean operator “AND” with infection‐related keywords (e.g., infectious diseases, infections, viral, bacterial, fungal and parasitic infections).

Given the narrative nature of the review, formal systematic‐review procedures (e.g., duplicate screening, predefined protocol registration, quantitative risk‐of‐bias assessment) were not performed. However, the following eligibility principles guided study selection:

- Population: Human studies were prioritised.
- Age group: Due to the limited availability of paediatric data, studies involving children and adolescents (< 18 years) were preferentially considered. When paediatric evidence was insufficient for specific endocrine systems, adult studies (> 18 years) were included to provide contextual and translational insights, acknowledging that findings from adult cohorts may not be directly generalised to paediatric populations due to differences in endocrine maturation, immune response and tissue susceptibility.
- Study design: Priority was given to meta‐analyses, systematic reviews, narrative reviews and original observational or interventional studies. Case series and selected case reports were included when higher‐level evidence was scarce or when they provided clinically relevant insights (e.g., rare but severe endocrine complications).
- Experimental evidence: Animal and in vitro studies were included when they contributed mechanistic or translational understanding relevant to endocrine dysfunction.

Publications not addressing endocrine outcomes as a primary or clearly defined secondary focus, non‐scientific reports and studies lacking sufficient methodological detail were excluded.

For each endocrine gland/system, findings were qualitatively summarised and interpreted to provide an integrated overview of both acute and chronic endocrine alterations associated with infectious diseases. The synthesis aimed to: highlight well‐established associations supported by higher‐level evidence; integrate mechanistic and clinical data where appropriate; identify inconsistencies across studies; delineate areas where evidence remains limited or absent.

As a narrative synthesis, this review emphasises interpretative integration of heterogeneous evidence. Potential limitations inherent to narrative reviews, including selection bias and variability in study quality, are acknowledged.

## 3 Hypothalamus and Pituitary Gland

Hypopituitarism is a disorder that results from the partial or complete reduction in the production of one or more hormones by the anterior and/or posterior lobe of the pituitary gland. Despite showing a growing trend in incidence and prevalence, it is a rare condition and often underdiagnosed due to its complexity and insidious onset [9].

The clinical manifestations of pituitary hormone deficiencies vary depending on the age at onset, the number of hormonal axes involved and the underlying aetiology. Whereas the most frequent causes of hypopituitarism are the compressive effect or surgical resection of hypothalamic or pituitary masses, a wide range of less frequent etiologies must be considered, among them genetic mutations, hypophysitis, traumatic brain injury, infiltrative and autoimmune diseases, vascular events and infections [10].

Infectious causes of hypopituitarism are particularly underrecognised and underexplored. The pituitary gland may be involved either by direct infection or by secondary dissemination from adjacent or distant sites. Mechanisms of involvement include direct exposure during neurosurgical procedures, local extension from contiguous structures, or hematogenous spread. Damage can arise not only from the infection itself but also from the immune and inflammatory responses it activates. In addition, any immunocompromised state represents a predisposing factor to be considered (e.g., diabetes mellitus, Cushing's syndrome, HIV infection, solid organ transplants, malignancies) [11].

Various pathogens including bacteria, viruses, fungi and parasites (Table 1) exhibit a tropism for brain tissue and can cause encephalitis, meningitis, meningoencephalitis, or brain abscesses. In the course of a central nervous system (CNS) infection, partial or complete hypothalamic–pituitary insufficiency may develop either transiently or as a permanent condition. Endocrine complications can present acutely or may develop insidiously over time, suggesting that long‐term monitoring is essential [4].

**TABLE 1:** Infectious agents and infections that can cause pituitary dysfunction.

Pituitary dysfunction	
Bacteria	*Mycobacterium tuberculosis*
	*Treponema pallidum*
	*Escherichia coli*
	*Streptococcus* group A
	*Streptococcus* group B
	*Streptococcus pneumoniae*
	*Haemophilius influenzae*
	*Neisseria meningitidis*
	*Staphylococcus aureus*
Viruses	Enterovirus
	HSV
	Sars Cov2
	Zika virus
	Coxsackie
	CMV
	Hantavirus
Fungi	*Aspergillus*
	*Candida*
	*Pneumocystis jirovecii*
	*Nocardia*
Parasites	*Toxoplasma gondii*

Evidence from clinical studies remains limited and heterogeneous. In one cohort of 19 patients recovering from CNS infections, six were found to have endocrine abnormalities, most notably, isolated ACTH deficiency and subclinical gonadotropin insufficiency [4]. Another study reported a 28.6% prevalence of isolated GH deficiency in individuals assessed 20 months post‐infection [12]. Similarly, in a separate cohort followed up at 6 and 12 months after meningitis, GH deficiency was the most commonly observed deficiency, reinforcing the need for extended follow‐up in adults who have experienced CNS infections [13]. These findings were confirmed in further studies [14]. In contrast, data from paediatric patients are more reassuring but not definitive. Two early studies on children recovering from CNS infections (involving 19 and 14 patients, respectively) did not report any endocrine abnormalities, concluding that routine monitoring of growth and pubertal development was sufficient [6, 15]. However, a subsequent investigation in 37 children with a history of bacterial meningitis confirmed a low rate of post‐infectious endocrine impairments, including GH deficiency in 3 patients. This would suggest a lower vulnerability of the pituitary gland to damage during central infections in children compared to adults, although there are few studies on this topic and findings are sometimes conflicting suggesting that paediatricians should be cautious under these circumstances [16].

Overall, it is hypothesised that inflammation due to infections may trigger autoimmune processes intensifying and prolonging neuroinflammation. The role of autoimmunity would be confirmed by the detection of elevated levels of autoantibodies (anti‐pituitary antibodies (APA) and anti‐hypothalamus antibodies (AHA)) in patients with late‐onset hypopituitarism following CNS infections, supporting a potential autoimmune pathogenesis [13]. Yet, the exact role and temporal dynamics of these antibodies remain unclear, and their presence is not routinely assessed in clinical practice.

Inappropriate secretion of antidiuretic hormone (SIADH) is a known complication of infections, in particular of the CNS. SIADH has been documented during various viral (e.g., HSV, enteroviruses, tick‐born virus) and bacterial infections (e.g., *Escherichia coli* , *Streptococcus pneumoniae* ) [17] (Table 2). SIADH is thought to be due to improper cellular activation induced by proinflammatory cytokines leading to non‐osmotic release of vasopressin and subsequent hyponatremia [18]. The severity of hyponatremia appears to be influenced by the causative pathogen, the extent of the infection and the involvement of specific brain regions [19]. However, whether SIADH in this context represents a transient parainfectious phenomenon or a marker of long‐term hypothalamic–pituitary dysregulation remains an open question.

**TABLE 2:** Infections and infectious agents that can lead to the Syndrome of Inappropriate Antidiuretic Hormone Secretion (SIADH).

SIADH	
Bacteria	*Escherichia coli*
	*Streptococcus pneumoniae*
Viruses	HSV
	Enterovirus

### 3.1 Bacterial Infections

Tuberculosis is a mycobacterial infection that, in its miliary form, can infiltrate the hypothalamus, the pituitary gland, or the infundibulum. The prevalence of central nervous system tuberculosis among patients with tuberculosis is approximately 4.55% [20]. Pituitary stalk thickening and tuberculomas have been documented on Magnetic Resonance Imaging (MRI). The recognition of this condition and the initiation of antitubercular therapy may allow normalisation of hormonal dysfunction and regression of pituitary tuberculomas [21]. In tuberculous meningitis, hyperprolactinemia, adrenal insufficiency, and central hypothyroidism have been reported, with nearly 30% of patients presenting with multiple hormone deficiencies. Diabetes insipidus appears to be rare [22]. In childhood, endocrine dysfunctions may present years later. One study reported 6/49 subjects having isolated hormone deficiencies, 3/49 having combined GH‐gonadotropin deficiencies, and none having diabetes insipidus. In 5/49 patients, the results of stimulation tests suggested a hypothalamic origin of the disorder [23]. In conclusion, in endemic areas and in patients with a history of tuberculosis, it is advisable to investigate tubercular involvement of the hypothalamic–pituitary region and assess hormonal function to treat appropriately and prevent permanent damage.

Syphilis is a sexually transmitted infectious disease that can affect the CNS and rarely presents with hypopituitarism. Neurosyphilis can develop during a primary syphilis infection in approximately 1.2% to 1.8% of cases [24] and can cause hypophysitis with subsequent endocrine dysfunctions as described in two case reports [25, 26]. However, neurosyphilis can also present as a late complication of chronic syphilis and present with hypopituitarism [27]. Neurosyphilis must still be considered in the differential diagnosis of endocrinological dysfunction, especially in the presence of risk factors.

Group B *Streptococcus (GBS)* can cause severe CNS infections in newborns. Antibiotic prophylaxis in women with positive vaginal swab screening is effective in preventing early‐onset sepsis in newborns but has no impact on late‐onset infections [28]. Hypopituitarism has been reported during severe GBS infection: patients presented initially with diabetes insipidus followed by TSH, ACTH and GH deficiencies leading to a significant deterioration of clinical conditions [29].

Pituitary abscesses, though uncommon (they represent less than 1% of pituitary lesions), have been documented in both adults and children, often mimicking adenomas. Bacteria are predominantly involved, including *Streptococci* and *Staphylococci*, as well as *E. coli* , *Mycobacteria* infections, *Neisseria meningitidis* and anaerobes [30, 31]. In children, pituitary abscesses can be associated with infections of the Rathke's cleft cyst. Surgical drainage and antibiotics may lead to partial or complete endocrine recovery, although some deficiencies may persist [32].

In conclusion, hypothalamic–pituitary dysfunction, although rare, is becoming increasingly epidemiologically relevant mainly because of infectious meningitis [12, 33]. Further studies will be needed to fully understand the extent of the phenomenon and potentially create appropriate surveillance protocols.

### 3.2 Viral Infections

#### 3.2.1 Viral Infections May Also Affect Hypothalamic and Pituitary Function

A good example is provided by the effects of hantavirus infection. These are transmitted by rodents and can cause haemorrhagic fever with a renal syndrome (HFRS). In China, which accounts for approximately 90% of the global burden of disease, the annual incidence ranges from 0.2 to 8 cases per 100,000 people from the general population [34]. In one study, in 60 adults who recovered from HFRS, 6 patients presented one single pituitary hormone deficiency and 5 subjects had multiple pituitary hormone deficiencies. Endocrine dysfunction rarely presented during the acute phase of the infection and usually presented later [35] being rarely persistent [36].

The COVID‐19 pandemic has intensified research into post‐viral endocrine complications. SARS‐CoV‐2 may reach the pituitary gland initially by binding to ACE2 receptors in the nasopharyngeal epithelium, subsequently entering the bloodstream, crossing the blood–brain barrier and ultimately spreading to the anterior pituitary [37, 38]. Pituitary dysfunction may not be solely attributable to direct cytopathic effects, as several alternative mechanisms have been proposed including inflammatory over‐activation of the hypothalamic–pituitary axis, immune‐mediated glandular injury (hypophysitis) and blood–brain barrier disruption secondary to an intracranial cytokine storm. Pituitary apoplexy has been reported following SARS‐CoV‐2 infection, occurring as early as 10 days after the onset of COVID‐19 symptoms or with a latency of up to 2 months. Its onset has been attributed to an imbalance between increased metabolic demand and reduced blood supply, further exacerbated by coagulopathy and thrombocytopenia [39]. Central adrenal insufficiency may present during both the acute phase and the post‐infectious recovery period, mainly in patients with severe infection [40]. However, the large use of corticosteroids in the management of COVID‐19 infections concerns about possible iatrogenic suppression of the hypothalamic–pituitary–adrenal axis, thereby complicating the interpretation of adrenal dysfunction in this context [37]. Other pituitary axes may also be affected. Mild and transient forms of central hypothyroidism have been described [41] and isolated growth hormone (GH) deficiency has been reported, typically between three‐and‐7 months post‐infection. Data on prolactin dynamics remain sparse, and the clinical significance of possible alterations is unclear. Hypogonadotropic hypogonadism has been observed [42, 43], with some cases suggesting subclinical or reversible forms in males [44, 45] and one report describing persistent secondary amenorrhea in a woman with no other identifiable cause aside from SARS‐CoV‐2 infection [46].

Of particular note, a rare case of a 4‐year‐old girl developing a phenotype resembling ROHHAD (Rapid‐onset Obesity with Hypothalamic Dysfunction, Hypoventilation and Autonomic Dysregulation) following COVID‐19 raises questions about the virus's potential to trigger or unmask complex hypothalamic syndromes [47].

In a deeper exploration of the paediatric context, an etiological study was conducted in a sample of 73 children with diabetes insipidus, which showed that in 11% of cases, the onset occurred after a CNS infection, sometimes caused by unspecified viruses as well as by different bacteria including *Streptococcus pyogenes* , *Haemophilius influenzae* and *Streptococcus pneumoniae* [48]. Central diabetes insipidus is reported to occur also secondary to encephalitis caused by Coxsackie B1 virus and has presented in newborns with congenital cytomegalovirus (CMV) infection [49, 50]. Congenital Zika infection has been described to be associated with central adrenal insufficiency and diabetes insipidus [51]. CMV has been identified as an infectious agent that can affect the hypothalamic areas that regulate vasopressin secretion leading to the onset of diabetes insipidus in a patient with Acquired Immune Deficiency Syndrome (AIDS) [52].

### 3.3 Fungal Infections

Fungal infections are rare in the immunocompetent population. Pituitary involvement has been occasionally reported. Fungi can spread through the hematogenous route, from the paranasal sinuses, from foci of osteomyelitis, through a cerebrospinal fluid fistula or as a complication of transsphenoidal surgical procedures [53].

An immunocompromised state can predispose to the formation of pituitary abscesses. Although these are usually of bacterial origin as previously described, *Aspergillus*, *Candida*, *Pneumocystis jirovecii* and *Nocardia* infections can occur also [54, 55]. Most commonly the infection occurs in the neurohypophysis, but the adenohypophysis can also be involved with subsequent panhypopituitarism or isolated hyperprolactinemia, hypogonadism, or rarely ACTH deficiency [54, 56, 57]. Rarely, abscesses are found in immunocompetent subjects [58].

Fungal involvement of the pituitary gland may occur not only via direct infection but also secondarily, through inflammatory erosion of the sellar floor in allergic fungal sinusitis, leading to mechanical compression and potential glandular dysfunction [59]. Treatment generally involves a combined surgical and medical approach yielding favorable results [60, 61].

### 3.4 Parasitic Infections

#### 3.4.1 Among Parasites, the Following Can Be Considered Based on the Findings in the Literature

*Toxoplasma gondii*, a protozoan with a high tropism for the CNS, can cause neurological infections [62], although there are few reports of hypopituitarism secondary to parasitic infections in patients with congenital infections or immunodeficiency [62, 63, 64]. In some cases, isolated deficiencies as for antidiuretic hormone evolved to panhypopituitarism over time [65, 66]. In most cases, antiparasitic therapy can lead to remission of endocrine dysfunction.

An interesting association of *T. gondii* pseudocysts and prolactin‐secreting pituitary adenomas has been described in two patients. Prolactin promotes the activation of antiprotozoal mechanisms in glial cells as a defensive response, suggesting that chronic antigenic stimulation of lactotrophs during infection may contribute to neoplastic transformation [67, 68]. A further case reported *T. gondii* bradycysts in a patient with a non‐secreting pituitary adenoma. Further studies are needed to understand the oncogenic potential of this parasite [69].

In summary, although emerging evidence links bacterial, viral, fungal and parasitic infections to hypothalamic–pituitary dysfunction, current data remain limited by small sample sizes, retrospective designs, heterogeneous diagnostic criteria and variable timing of endocrine assessment. A crucial unresolved issue is the distinction between transient adaptive neuroendocrine responses during acute or critical illness and irreversible structural pituitary damage. Hormonal alterations observed in the acute phase of CNS infections may reflect physiological stress responses, cytokine‐mediated modulation of hypothalamic signalling, or effects of therapeutic interventions, particularly glucocorticoids, rather than permanent glandular injury. Similarly, abnormalities detected months after infection must be interpreted cautiously in the absence of baseline endocrine evaluation or standardised dynamic testing. Confounding factors, including critical illness‐related corticosteroid insufficiency, non‐thyroidal illness syndrome, exogenous steroid exposure and selection bias in patients undergoing neuroimaging or endocrine testing, further complicate causal interpretation. In this context, SIADH often represents a transient parainfectious phenomenon, whereas persistent diabetes insipidus or confirmed anterior pituitary deficits require thorough reassessment over time.

The variability in reported prevalence rates likely reflects differences in study design, diagnostic thresholds and follow‐up duration rather than true epidemiological differences. Particularly in paediatric populations, long‐term consequences remain insufficiently characterised. Future research should prioritise large prospective multicentre studies incorporating standardised dynamic stimulation testing protocols, clearly defined biochemical diagnostic criteria, serial hormonal reassessment and long‐term longitudinal follow‐up.

Only through methodologically rigorous approaches will it be possible to distinguish adaptive neuroendocrine dysregulation from permanent endocrine failure and to define appropriate surveillance and management strategies.

## 4 Thyroid Gland

The thyroid gland has a notable resistance to infections. The low incidence of thyroid infections is thought to result from multiple protective factors, including its elevated iodine content, robust vascular and lymphatic circulation, and the presence of a capsula that anatomically excludes the gland from adjacent cervical structures [70]. Despite these protective features, infections of bacterial, viral, parasitic or fungal origin can still involve the thyroid gland leading to a range of clinical conditions, such as acute suppurative thyroiditis (AST), subacute thyroiditis, or even trigger autoimmune thyroid disorders (Table 3).

**TABLE 3:** Infectious agents and infections that can cause thyroid infections.

Thyroid infections	
Bacteria	*Staphylococcus aureus*
	*Streptococcus pyogenes*
	*Streptococcus epidermidis*
	*Streptococcus pneumoniae*
Viruses	*Human foamy virus*
	*Mumps virus*
	*Ebstein Bar virus*
	*Cytomegalovirus*
	*SARS‐Cov‐2*
	*Human T.cell Lymphotropic Viruss Type 1*
	*Human immunodeficiency virus*
	*Simian virus 40*
	*Rubella virus*
	*Herpes simplex virus*
	*Hepatitis C virus*
	*Parvovirus B19*
	*Enterovirus*
Fungi	*Aspergillus* spp.
	*Candida* spp.
	*Cryptococcus*
	*Histoplasma*
	*Pseudallescheria*
	*Blastomyces* spp.
	*Scedosporium* spp.
	*Coccidioides* spp.
Parasites	*Toxoplasma gondii*
	*Nippostrongylus*
	*Enatamoeba hystolitica*
	*Echinococcus*

In addition, transient dysregulation of thyroid hormone levels may occur in the setting of critical systemic illnesses, including severe infections, without intrinsic thyroid pathology. This phenomenon, known as non‐thyroidal illness syndrome (NTIS), is characterised by reduced T3 and free T3 and normal or decreased thyroid‐stimulating hormone (TSH). Recent studies report the prevalence of NTIS in up to 70% of paediatric intensive care unit (PICU) patients with sepsis [71]. The pathophysiology involves several factors: reduced peripheral conversion of T4 to T3, activation of inflammatory cytokines such as TNF‐α and IL‐6, increased oxidative stress and decreased expression of thyroid hormone receptors. NTIS does not require thyroid hormone replacement, being a transient condition; however, long‐term data on thyroid function are limited, and there is currently no consensus regarding follow‐up strategies to identify persistent dysfunction [72].

### 4.1 Bacterial Infections

Bacterial infections, although uncommon, can have serious clinical consequences in paediatric populations. AST represents the most frequently observed bacterial thyroid condition in children, typically occurring in those with underlying congenital anomalies, most notably piriform sinus fistula (PSF) [71, 73, 74, 75, 76, 77]. PSF creates an abnormal communication between the pharynx and thyroid, usually on the left side. In a series of 17 paediatric patients (mean age 9.6 years), PSF was identified in 58% of cases, presenting with recurrent thyroid abscesses, fever, pain and unilateral neck swelling [78]. Diagnostic approaches such as barium swallow and CT imaging detected the fistula in up to 88% of patients, while fine‐needle aspiration enabled microbial identification and guided targeted antibiotic therapy. The most commonly isolated organisms are *Staphylococcus aureus* and *Streptococcus* spp., frequently in mixed infections [77, 79]. Definitive long‐term resolution generally requires surgical or endoscopic intervention in combination with drainage and antibiotic treatment [80, 81, 82].

Diagnosis of thyroid involvement requires a multidisciplinary approach. AST is evaluated through careful clinical history, physical examination, thyroid ultrasound, barium esophagogram to detect PSF, and fine‐needle aspiration for pathogen identification [71, 78, 82, 83].

### 4.2 Viral Infections

Viral infections are considered potential environmental contributors to subacute thyroiditis and autoimmune thyroid disorders [84, 85]. However, given the high background prevalence of common viral exposures in the general population, establishing a direct causal relationship remains challenging.

Several observations support a possible association between viral infections and subacute thyroiditis, including its seasonal distribution, frequent onset after upper respiratory tract infections, and preceding viral‐like symptoms [86, 87, 88]. Nevertheless, these features are not specific and do not establish causality. Thyroid dysfunction during or after infection may reflect nonspecific immune activation rather than direct viral injury.

Proposed mechanisms include immune‐mediated inflammation, lymphocytic responses to viral antigens, antigen release from damaged thyrocytes, molecular mimicry, and immune activation via HLA‐DR expression or Toll‐Like Receptors (TLRs) [88, 89, 90]. While biologically plausible, these hypotheses derive largely from experimental or associative data and lack consistent confirmation in controlled human studies.

Desailloud and Hober reported the detection of viral components in thyroid tissue, including HFV and mumps virus in subacute thyroiditis; HTLV‐1, HFV, HIV and SV40 in Graves' disease; and HTLV‐1, enteroviruses, EBV and parvovirus B19 in Hashimoto's thyroiditis. Although these findings suggest a possible link, several methodological limitations must be considered. Detection techniques were heterogeneous, often lacked standardised validation, and were susceptible to contamination. Highly sensitive PCR‐based methods may detect latent or incidental viral nucleic acids without demonstrating active infection or pathogenic relevance. Moreover, viral material identified in inflamed thyroid tissue may reflect bystander immune cell infiltration rather than primary thyroid infection. Importantly, consistent replication of these findings across independent cohorts has not been uniformly achieved [88].

A more recent review evaluating viruses such as foamy virus, parvovirus B19, and EBV in Graves' disease similarly reported inconclusive and inconsistent results [91], further underscoring the need for cautious interpretation.

In hepatitis C virus (HCV) infection, an association with thyroid autoimmunity and hypothyroidism has been described [92]. However, whether this reflects direct viral invasion, immune dysregulation, treatment‐related effects (e.g., interferon therapy), or shared susceptibility remains incompletely clarified. Thyroid dysfunction is considered an extrahepatic manifestation of HCV infection, and thyroid function testing is recommended in symptomatic patients [93].

Although data in children are limited, viral infections have also been implicated in paediatric thyroid dysfunction. Case reports describe EBV‐related hypothyroidism and subacute thyroiditis [94, 95], CMV‐associated thyroiditis [96], and thyroid dysfunction in congenital Zika virus infection [97]. However, isolated case reports cannot establish causality, and transient thyroid alterations must be distinguished from persistent autoimmune disease. Similarly, thyroid autoimmunity observed in congenital rubella syndrome may emerge years after infection, but the contribution of background autoimmune susceptibility remains uncertain [98, 99].

Thyroid abnormalities have also been described in perinatally acquired HIV infection [100]. Subclinical hypothyroidism, isolated low FT4, and euthyroid sick syndrome are common and may represent adaptive responses to chronic illness rather than intrinsic thyroid pathology. Graves' disease may occur in the context of immune reconstitution inflammatory syndrome (IRIS) after antiretroviral therapy initiation [101], highlighting the complex interplay between immune recovery and autoimmunity rather than a direct viral effect.

The global spread of SARS‐CoV‐2 has intensified interest in viral‐related thyroid dysfunction. A 2024 systematic review reported thyroid abnormalities in approximately 15% of infected adults [102]. However, many reported alterations, such as non‐thyroidal illness syndrome (NTIS), likely reflect systemic illness rather than primary thyroid involvement. Although direct viral entry via ACE2 receptors and immune‐mediated mechanisms have been proposed, current evidence does not demonstrate increased susceptibility to SARS‐CoV‐2 infection in individuals with pre‐existing thyroid disease [103]. Subacute thyroiditis appears to be an uncommon complication, and most cases resolve spontaneously. Importantly, longitudinal follow‐up studies up to 2 years have not demonstrated persistent, clinically significant thyroid dysfunction directly attributable to SARS‐CoV‐2 [102].

Overall, while numerous studies report associations between viral infections and thyroid dysfunction, including subacute thyroiditis, autoimmune thyroid disease, and transient NTIS, the evidence remains predominantly observational. Reverse causation, background viral exposure, nonspecific immune activation, and methodological heterogeneity limit causal inference. Detection of viral components in thyroid tissue does not necessarily imply pathogenic relevance and may reflect latent infection or technical artefacts. Many proposed mechanisms, such as molecular mimicry and TLR‐mediated activation, remain hypothetical and require validation in large, prospective, well‐controlled studies with standardised viral detection protocols [104, 105, 106, 107, 108].

Distinguishing transient adaptive responses from persistent autoimmune or structural thyroid disease is essential, particularly in paediatric populations and in emerging infections. Future research should prioritise longitudinal, replicable, and methodologically rigorous investigations to clarify the true role of viral infections in thyroid autoimmunity and to inform evidence‐based screening strategies.

### 4.3 Fungal Infections

Fungal thyroiditis, despite its general rareness, is the second cause of thyroid infections [108]. The literature provides a series of cases regarding immunocompromised people, particularly patients being treated for blood neoplasms, organ transplant, AIDS, iatrogenic immunosuppression, or autoimmune diseases.

Although many patients don't present specific symptoms or laboratory markers, some develop nonspecific signs of acute thyroiditis (i.e., fever, swallowing and tenderness of the neck, dysphagia, dysphonia) and thyroid dysfunction (i.e., hyperthyroidism in the early phases then followed by transient or permanent hypothyroidism) [108].

In a 2006 review [108] several cases of thyroid infections caused by *Aspergillus*, *Candida*, *Cryptococcus*, *Coccidioides*, *Histoplasma* and *Pseudallescheria* were reported. A more recent review identified in addition *Blastomyces* and *Scedosporium* [79]. *Mucorales* is another fungus described rarely in the literature: only 11 cases have been reported, and among these, one was a child having lymphoblastic leukaemia [109].

*Aspergillus* is the most common fungus causing thyroiditis, and in most cases, patients have a disseminated infection. Often thyroid invasion is only an autopsy finding and thyroid function is usually normal [108]. In children, as in adults, this infection is associated with immunocompromised conditions such as in granulomatosis diseases [110] and renal transplantation [111, 112]. Goldani et al. [108] reported the case of a fatal disseminated infection in a newborn with erythroblastosis fetalis [113]. Only one case of a systemic *Aspergillus* infection concerned a previously healthy child (18 days old), but in a condition of para‐physiological immunosuppression due to neonatal age [114]. Among case‐reports on *Candida* infections [108], only one concerned a 17‐year‐old boy who developed a thyroid abscess after fungal blood spreading in leukaemia related neutropenia. This adolescent developed permanent hypothyroidism after surgical and medical treatment [115].

With regard to *Cryptococcus*, *Coccidioides*, *Histoplasma* and *Pseudallescheria* infections, no paediatric cases have been reported in the literature [108].

### 4.4 Parasitic Infections

There is scarce evidence of parasitic infections of the thyroid gland in the literature. Lafontaine et al. reported *T. gondii* and *Nippostrongylus* to be causes of suppurative thyroiditis in immunocompromised people [79]. In particular, *T. gondii* latent infection was associated with the development of gestational hyperthyroidism in pregnant women [116]. According to more recent studies, *T. gondii* stimulates thyroid function, and this in turn may trigger the production of autoantibodies against thyroid peroxidase, leading to the development of autoimmune hyperthyroidism [117].

*Entamoeba histolytica* is described as a cause of suppurative thyroiditis in endemic areas, being extremely rare in the general population [85]. Infrequently, *Echinococcus* infections can localise in the thyroid gland where the parasite proliferates, forming cysts within the gland. Patients with this condition sought medical attention for the appearance of a thyroid goitre with no presenting symptoms related to thyroid dysfunction [118].

## 5 Adrenal Glands

Hypocortisolism is a potentially life‐threatening condition requiring prompt recognition and treatment. It may result from impaired hypothalamic or pituitary function (central or secondary adrenal insufficiency) or from primary adrenal gland destruction (primary adrenal insufficiency [PAI], also known as Addison's disease) [119]. Distinguishing between these entities is essential, as pathophysiology, prognosis and long‐term management differ substantially.

In children, PAI is predominantly associated with genetic etiologies, accounting for approximately 70%–85% of cases, while autoimmunity represents the second most common cause [120]. The contribution of infectious diseases to PAI varies geographically. In regions with a high prevalence of tuberculosis and systemic infections, such as parts of Asia and sub‐Saharan Africa, infections remain a significant cause [121]. In contrast, in high‐income countries, infectious etiologies are uncommon, with tuberculosis representing a minority of cases (approximately 9% in one large series) [122]. In paediatric populations, infection‐related PAI is described mainly in isolated reports, and reliable prevalence data are lacking.

Several pathogens (Table 4) exhibit tropism for the adrenal glands, where inflammation, haemorrhage, granulomatous infiltration, or necrosis may impair steroidogenesis. However, clinically overt Addison's disease typically manifests only after destruction of approximately 80%–90% of the adrenal cortex [123]. Importantly, radiological or biochemical abnormalities do not necessarily equate to permanent adrenal failure.

**TABLE 4:** Infections and infectious agents causing adrenal dysfunction.

Adrenal dysfunction	
Bacteria	*Mycobacterium tuberculosis*
	Atypical *Mycobacteria*
Viruses	Hepatitis B virus
	Hepatitis C virus
	Herpes virus 6
	SARS‐Cov‐2
	Human immunodeficiency virus
	Cytomegalovirus
	Epstein–Barr virus
Fungi	*Cryptococcus neoformans*
	*Blastomyces dermatitidis*
	*Histoplasma capsulatum*
	*Coccidioides immitis*
	*Paracoccidioides brasiliensis*
	*Pneumocystis jirovecii*
Parasites	*Toxoplasma gondii*
	*Trypanosoma cruzi*
	*Leishmaniosis*

A critical distinction must be made between structural PAI and adrenal dysfunction occurring during acute systemic illness. In critically ill patients, cortisol dynamics are complex and influenced by altered binding proteins, cytokine‐mediated modulation of the hypothalamic–pituitary–adrenal (HPA) axis, and tissue‐level glucocorticoid resistance. Critical illness‐related corticosteroid insufficiency (CIRCI) reflects a dysregulated stress response rather than intrinsic adrenal destruction [124].

According to Society of Critical Care Medicine (SCCM) and European Society of Intensive Care Medicine (ESICM) guidelines, CIRCI may be suggested by a rise in cortisol < 9 μg/dL after a 250 μg ACTH stimulation test or a random plasma cortisol < 10 μg/dL. By contrast, PAI is typically defined by a peak cortisol < 18 μg/dL after ACTH stimulation. However, these thresholds overlap, creating a diagnostic ‘grey zone’. Moreover, reliance on single random cortisol measurements during acute infection is problematic, as values may reflect adaptive physiological responses rather than true adrenal failure. For this reason, biochemical findings in the acute phase should be interpreted cautiously and, when feasible, reassessed after clinical stabilisation before establishing a diagnosis of permanent adrenal insufficiency.

It is also important to distinguish transient biochemical hypocortisolism from clinically significant adrenal insufficiency requiring lifelong replacement therapy. In some infectious contexts—particularly tuberculosis and certain fungal infections—adrenal dysfunction may partially or fully recover following appropriate antimicrobial therapy. Conversely, extensive bilateral adrenal destruction may lead to irreversible PAI. The extent to which antifungal and antitubercular treatments facilitate adrenal recovery remains variable and incompletely characterised, underscoring the need for longitudinal endocrine reassessment after infection resolution.

Overall, accurate stratification between primary adrenal destruction, secondary/central dysfunction, and stress‐related adaptive alterations is essential to avoid overestimating the burden of infection‐related adrenal disease and to guide appropriate long‐term management strategies.

### 5.1 Bacterial Infections

*Mycobacterium tuberculosis* is a prevalent cause of adrenalitis where the infection is endemic. Approximately one third of patients with active tuberculosis may develop adrenal insufficiency [125]. In the adrenal glands, *M. tuberculosis* causes calcification foci that represent the most frequent radiological feature of this condition [126, 127]. In some cases, the function of the adrenal gland may be restored after appropriate eradication therapy [127]. In the literature, one can find a well‐described association between bilateral involvement of adrenal glands by tuberculosis and co‐infection by HIV virus [128]. In addition, adrenal gland infiltration by the atypical *Mycobacterium avium* has been described in patients with AIDS [123, 127]. Nevertheless, in the paediatric population, tuberculous adrenalitis presenting with acute adrenal insufficiency has been reported only anecdotally, mainly as isolated case reports, highlighting the rarity of this presentation in children [129, 130, 131].

Adrenal insufficiency in tuberculosis can be caused by direct tissue destruction due to pathogen invasion, but it can be related to the toxicity of rifampicin, one of the major antibiotics used for its treatment. Rifampicin is a potent inducer of hepatic cytochrome P450 enzyme, leading to accelerated metabolism of endogenous and exogenous glucocorticoids and potentially resulting in functional adrenal insufficiency, particularly in patients with limited adrenal reserve or on steroid replacement therapy. Case reports and small series have described adrenal crises or increased glucocorticoid requirements in patients with tuberculosis during rifampicin treatment, with symptoms resolving after dose adjustment or drug discontinuation [132, 133].

Bilateral adrenal haemorrhage, resulting in a dangerous condition called Waterhouse Friderichsen syndrome, can have a fatal outcome in approximately 15%–50% of the cases, but when it has a favourable course, it results in permanent adrenal insufficiency, rarely in transient deficiency [134]. It is secondary to potentially fatal clinical situations, such as disseminated infections and sepsis. Indeed, animal studies have proven that adrenal haemorrhage can be triggered by inoculation of bacterial endotoxins into the venous system only after administration of ACTH, as if in stressful situations the elevated metabolic activity linked to corticosteroids production renders the adrenal cortex more susceptible to bleeding. Furthermore, endotoxins contribute to adrenal haemorrhage mediating the activation of coagulation and fibrinolysis, besides the production of inflammatory molecules [135]. The most frequent bacteria causing adrenal haemorrhage are *N. meningitidis*; other agents described are *Enterobacter cloacae*, *H. influenzae*, *S. aureus*, *Corynebacterium diphtheriae*, *S. pneumoniae*, *Neisseria gonorrheae*, *Streptococcus* group A, *Klebsiella oytoca* and *Pseudomonas aeruginosa* [136, 137] (Table 5).

**TABLE 5:** This table shows the list of pathogens that can cause Waterhouse Friderichsen Syndrome.

Waterhouse Friderichsen Syndrome	
Bacteria	*Streptococcus* group A
	*Streptococcus pneumoniae*
	*Haemophilius influenzae*
	*Neisseria meningitidis*
	*Neisseria gonorreae*
	*Enterobacter cloacae*
	*Klebsiella oxytoca*
	*Pseudomonas aeruginosa*
	*Corynebacterium diphtheriae*
	*Staphylococcus aureus*
Viruses	Echovirus 6
	Varicella Zoster virus

### 5.2 Viral Infections

In adult patients with AIDS, Addison disease can be secondary to CMV infections [127], being described also in other conditions of immune vulnerability [126]. Little is known in paediatric patients. What has emerged from a study of the current literature is that viral adrenalitis, resulting in adrenal insufficiency, has been rarely described in immunocompetent children.

Dinleyici et al. described two infants, a 28‐day‐old girl and a 59‐day‐old boy, presenting with lethargy and hypotension and a suggestive electrolytic pattern (hyperkalaemia, hyponatremia) with low cortisol levels and elevated serum ACTH levels. In both patients, serology and quantitative dosage of CMV‐DNA on blood were consistent for CMV infection. Antiviral therapy and steroids led to complete recovery of adrenal function in 1 week of treatment, and steroid therapy was tapered till discontinuation [138]. Another case report described a 32‐day‐old female who presented as lethargic and hypotensive, with low sodium, high potassium levels and enlarged adrenal glands. CMV and EBV co‐infection was proven and ganciclovir treatment started with subsequent recovery of adrenal function [139]. All these infants were interestingly below the age of 2 months, which leads to consideration that the immaturity of the immune system, typical of this age, represents a risk factor for adrenal involvement. Moreover, they all share a transient nature of the insufficiency and other causes of adrenal failure could be excluded supporting the hypothesis that the infection was the *primum movens* of these conditions.

Focusing on EBV infection, only one further case was reported in the literature in a 10‐year‐old boy with Wiskott‐Aldrich syndrome who was admitted with typical clinical and laboratory findings of PAI, and clinical‐serological evidence of EBV infection. In this patient, adrenal failure was permanent, confirmed by the ACTH‐stimulation test, and the patient underwent replacement therapy until he died from another infectious disease. This case differs from the others since this patient had a T‐cell immune‐deficiency syndrome, and the authors made two possible hypotheses on the causes: a cytopathic destruction of the adrenal cells mediated directly by EBV replication and/or B‐cell uncontrolled proliferation due to lack of T‐cell regulation with production of autoantibodies against adrenal cells [140].

One case of transient adrenal failure, confirmed by the ACTH‐stimulation test, was described in a 6‐year‐old boy with Adenovirus serotype 40 infection, proving that *Adenoviruses* have a tropism for the adrenal gland [141].

HBV and HCV virus infection have been reported in adult patients with adrenal insufficiency [126]. Schmitt et al. stressed the possibility that Herpesvirus‐6 infection could have triggered adrenal insufficiency, confirmed by ACTH‐stimulation test, in a 12‐month‐old girl presenting with jaundice and hepatomegaly, probably through an autoimmune mechanism [142].

Autoimmune diseases are known to develop in genetically predisposed individuals following environmental triggers, with viral infections frequently implicated. In autoimmune Addison's disease (AAD), this hypothesis is supported by seasonal incidence peaks, typically in winter and early summer, mirroring patterns of viral circulation. The adrenal cortex may serve as a viral reservoir, allowing for latent infections that would promote chronic inflammation over time. Additionally, several AAD‐associated genetic variants involve antiviral immune pathways, and patients often show impaired natural killer cell function, further suggesting a role for defective viral clearance in disease pathogenesis [126].

Multi‐system involvement by SARS‐Cov‐2 infection has been described. Several case reports of primary adrenal insufficiency triggered by this infection are present in the literature. A systematic review reported that the prevalence of adrenal insufficiency among patients with COVID‐19 varies widely, ranging from 3.1% to 64.3% across different studies. Nevertheless, most investigations have documented a prevalence below 10% [143]. We collected two case reports regarding children/adolescents. The first concerns an 18‐year‐old boy who developed AAD and myocarditis 10 days after developing acute respiratory distress syndrome [144]; the second describes hypothyroidism secondary to autoimmune thyroiditis and adrenal insufficiency presenting 3 weeks after SARS‐Cov‐2 infection [145]. In adults, acute adrenal insufficiency linked to SARS‐Cov‐2 infection is more frequent [143, 146]. However, it has not been possible to assess whether adrenal insufficiency in these cases is a form of PAI or a CIRCI subsequent to the severe stress induced by the infection [144].

With regard to viruses causing bilateral haemorrhage, we found only one case report of Waterhouse Friderichsen syndrome induced by varicella zoster virus (VZV) in an adult patient [147] and one case report of a neonate with multisystemic Echovirus 6 infection in whom adrenal haemorrhage was discovered after autopsy [148] (Table 5).

### 5.3 Fungal Infections

The relationship between fungal infections and endocrinological dysfunction is dual. Indeed, some endocrinological conditions such as Cushing disease, diabetes mellitus or Autoimmune Polyendocrine Syndrome‐1 predispose to systemic fungal infection and simultaneously fungi can invade glands [149]. The adrenal glands are the most frequently involved glands in generalised fungal infections [149]. Frenkel et al. suggested that this could be due to a local state of immunodepression generated by the high tissue concentration of glucocorticoids [150]. Since immunodepression facilitates fungal systemic diffusion, immunocompromised people are at higher risk of adrenal gland involvement when opportunistic microbes disseminate.

*Histoplasma capsulatum* and *Paracoccidioides brasiliensis* infections are the most frequently described in association with AAD [149]. In patients with disseminated histoplasmosis, adrenal involvement has been observed in 30%–50% of cases, with clinical adrenal insufficiency occurring in approximately 5%–50% of cases [123, 151]. Similarly, in paracoccidioidomycosis, adrenal involvement is found in 44%–80% of autopsy cases, and functional insufficiency is reported in 14%–44% of those with adrenal infection [152]. These data originate predominantly from case series and autopsy studies conducted in Latin America, Southeast Asia and parts of the United States.

There are also several reports of infections by *Pneumocystis jirovecii* [127], episodically described in a non‐immunocompromised adult patient [153], *Blastomyces dermatitidis*, *Coccidioides immitis* and *Cryptococcus neoformans* [126, 127].

Timely antifungal treatment is necessary in the attempt to avoid permanent adrenal insufficiency, even if in most cases, adrenal gland function does not recover [149].

### 5.4 Parasitic Infections

*Trypanosoma cruzi* infections, primarily reported in endemic regions such as Sub‐Saharan Africa and Latin America, and ubiquitous *T. gondii* infections have both been associated with adrenal failure in immunocompromised patients [126, 127]. Adrenal insufficiency has been described in 50% of patients with visceral leishmaniasis, in a Brazilian series [154].

In conclusion, while various pathogens can invade the adrenal glands, clinically overt adrenal insufficiency is uncommon, as over 80%–90% of adrenal tissue must be destroyed before dysfunction becomes apparent. Nonetheless, clinicians should maintain a high index of suspicion in patients with infections who present with signs such as refractory hypotension, fatigue, vomiting, abdominal pain, hyponatremia, hyperkalemia, low cortisol and elevated ACTH levels. This is particularly crucial in immunocompromised individuals. In the paediatric population, this review highlights the prominent role of viral infections in triggering adrenal dysfunction, especially in previously healthy neonates. Early identification of the infectious agent is essential for initiating targeted therapy and optimising adrenal recovery.

## 6 Gonads

Compared with other endocrine axes, the impact of infectious diseases on the gonadal axis remains insufficiently characterised. Available evidence is fragmented, predominantly derived from adult cohorts, and frequently limited to cross‐sectional studies, case reports, or narrative reviews. Causal inference is therefore limited. This section should be interpreted as reflecting a substantial knowledge gap, particularly regarding paediatric populations, pubertal development, fertility potential and long‐term reproductive health.

Hypogonadism may result from primary gonadal failure or secondary hypothalamic–pituitary dysfunction, biochemically distinguished by gonadotropin levels: elevated LH in primary hypogonadism versus inappropriately normal or low LH in secondary forms [155]. However, biochemical alterations do not necessarily equate to clinically significant reproductive impairment, especially in the context of acute or chronic systemic illness.

In paediatric and adolescent populations, pubertal timing and progression are key indicators of gonadal integrity. Chronic infections, most notably HIV, have been associated with delayed pubertal onset or slowed progression [156]. Nevertheless, much of the available evidence derives from observational or cross‐sectional studies, and confounding factors such as malnutrition, systemic inflammation, socioeconomic determinants, and antiretroviral therapy complicate interpretation. Although impaired Leydig cell function and secondary hypogonadism may theoretically affect spermatogenesis and future fertility [156], robust longitudinal studies assessing pubertal milestones, semen parameters, and adult fertility outcomes are largely lacking. Consequently, the long‐term reproductive implications of childhood or adolescent infections remain uncertain.

### 6.1 Bacterial, Fungal and Parasitic Infections

Direct infectious orchitis has been described in association with *Mycobacterium tuberculosis* [157] and *Treponema pallidum* [158, 159] However, evidence is largely limited to isolated case reports documenting epididymo‐orchitis or granulomatous testicular involvement. Systematic endocrine evaluation and longitudinal fertility follow‐up are generally absent, precluding conclusions regarding persistent hypogonadism or reproductive outcomes.

Parasitic infections such as visceral leishmaniasis and African trypanosomiasis are characterised by significant immune activation and systemic illness [160, 161]. Nonetheless, there are no well‐designed clinical studies demonstrating consistent long‐term gonadal endocrine impairment. Paediatric‐specific data addressing pubertal progression, testicular growth, ovarian function, or subsequent fertility in endemic regions are virtually nonexistent. This represents a critical and under‐recognised gap in global paediatric endocrine research.

Overall infectious causes of hypo‐ and hypergonadotropic hypogonadism are summarised in Tables 6 and 7, respectively.

**TABLE 6:** Bacterial and viral infections causing hypogonadotropic hypogonadism.

Hypogonadotropic hypogonadism	
Bacteria	*Mycobacterium tuberculosis*
	Atypical *Mycobacteria*
Viruses	Human immunodeficiency virus

**TABLE 7:** Bacterial and viral infectious agents causing hypergonadotropic hypogonadism.

Hypergonadotropic hypogonadism	
Bacteria	*Mycobacterium tuberculosis*
	Atypical *Mycobacteria*
	*Treponema pallidum*
Viruses	Human immunodeficiency virus
	Mumps virus
Fungi	*Cryptococcus* spp.
	*Blastomyces* spp.

### 6.2 Viral Infections

Overall viral infections that have been reported to cause hypo‐ and hypergonadotropic hypogonadism are summarised in Tables 6 and 7, respectively.

Among viral infections, HIV represents the most extensively studied model of infection‐associated hypogonadism, although important limitations remain.

In a contemporary cross‐sectional cohort of young and middle‐aged men receiving effective combination antiretroviral therapy, Lachâtre et al. reported a prevalence of biochemical hypogonadism of 8.7%, despite virologic suppression [156]. Earlier studies, largely conducted in the pre–combination therapy era and summarised in narrative reviews [155, 162], reported higher prevalence rates (13%–40%), suggesting that improved disease control may reduce but not eliminate the risk.

Secondary hypogonadism appears to be the predominant phenotype [155, 162], plausibly reflecting functional hypothalamic–pituitary suppression related to chronic inflammation, systemic illness, metabolic comorbidities, and medication effects rather than irreversible structural damage. Primary gonadal impairment has also been reported [156], though mechanisms remain incompletely understood. Importantly, most available data concern adult males, and paediatric‐specific evidence evaluating pubertal timing, gonadal maturation, spermatogenesis, ovarian reserve, or long‐term fertility is extremely limited.

Overall, while associations between infectious diseases and gonadal dysfunction have been described, current evidence is largely observational and insufficient to establish causality or quantify long‐term reproductive risk. Carefully designed prospective cohort studies, with standardised hormonal assessment, pubertal staging, and long‐term reproductive follow‐up are needed to clarify the true burden and clinical significance of infection‐related gonadal dysfunction, particularly in children and adolescents.

## 7 Endocrine Pancreas

It is widely recognised that infections can trigger autoimmune disorders, predominantly viral rather than bacterial infections [163]. Type 1 diabetes mellitus (T1DM) is a chronic autoimmune disease resulting from the selective destruction of pancreatic β‐cells. Its pathogenesis involves both genetic predisposition, particularly HLA‐DR genotypes (DR1, DR3, DR4, DR16), and environmental triggers, including viral infections, birthweight, early cow's milk intake, obesity and micronutrient deficiencies [164]. The global prevalence of T1DM is estimated at 9.5%, with significant geographic variability [164].

Despite extensive research, paediatric‐specific longitudinal data remain limited, leaving knowledge gaps regarding the timing of viral exposure, progression to autoimmunity and long‐term β‐cell outcomes.

### 7.1 Bacterial, Fungal and Parasitic Infections

Bacterial, fungal and parasitic infections have been increasingly studied as potential contributors to the onset or acceleration of T1DM in genetically susceptible individuals [165]. Bacterial antigens, for instance from *S. pneumoniae* , *M. tuberculosis* , or components of the gut microbiota, may cross‐react with β‐cell antigens, promoting autoimmunity [166]. Fungal dysbiosis, particularly *Candida albicans* colonisation, may modulate immune responses and skew cytokine production, contributing to a pro‐inflammatory environment that can influence autoimmunity [167]. Parasitic infections may impact T1DM pathogenesis through alteration of gut barrier integrity, modulation of Th1/Th2/Th17 balance, or stimulation of regulatory T cells [165].

While these mechanisms are supported by experimental models and some observational studies, robust human evidence remains scarce, particularly in paediatric populations [168, 169]. Thus, bacterial, fungal and parasitic triggers should be considered plausible but not yet conclusively proven contributors to T1DM pathogenesis.

### 7.2 Viral Infections

Viruses are the most consistently reported environmental triggers of T1DM [163, 170] (Table 8). Among these, enteroviruses, particularly Coxsackievirus B (CVB), have the strongest evidence for involvement in β‐cell autoimmunity. The median prevalence of enterovirus infection in paediatric populations is estimated at ~14.4% [171]. In vitro studies demonstrate that CVB isolates from newly diagnosed T1DM patients can destroy human β‐cells [172], and enteroviruses have been detected in intestinal biopsies of up to 75% of T1DM patients [173]. Furthermore, viral RNA has been identified in pancreatic islets and peripheral blood mononuclear cells in adults with T1DM [174, 175], suggesting a role in either initiation or acceleration of autoimmune β‐cell destruction. Proposed mechanisms include molecular mimicry, for example between the enteroviral 2C protein and glutamic acid decarboxylase (GAD), and activation of pre‐existing autoreactive T‐cells [163, 170].

**TABLE 8:** Viral Infections related with the onset of Type 1 diabetes mellitus.

Type 1 diabetes mellitus	
Viruses	Rotavirus
	Enterovirus
	SARS‐Cov‐2
	Influenza H1N1
	Cytomegalovirus
	Mumps virus

Other viruses have been investigated with varying levels of evidence. Rotavirus infection and vaccination have been studied for potential influence on T1DM onset. Mechanisms include direct pancreatic injury, molecular mimicry with islet antigen‐2 (IA‐2), and bystander activation of autoreactive T‐cells. Cohort studies, including BabyDiab in Australia and the DIPP project in Finland, yield mixed results: some studies found an association between rotavirus infection and autoantibody development, while others observed no significant impact, likely reflecting differences in study populations, methods, and viral strain exposure [176]. Human herpesviruses, including CMV, HSV, EBV and VZV, have also been implicated. CMV infection has been associated with higher islet autoantibody titers and accelerated diabetes onset in small human cohorts [177] and experimental models [178, 179], yet larger population‐based studies show inconsistent associations, highlighting uncertainty regarding causality [164]. Influenza H1N1 strains have been shown in vitro to replicate in pancreatic cells without inducing significant cytotoxicity, suggesting a modulatory rather than directly cytopathic effect [180]. SARS‐CoV‐2 has recently been reported to infect pancreatic β‐cells via alternative receptors, such as neuropilin‐1, leading to apoptosis and impaired insulin secretion, although whether this infection triggers T1DM or accelerates pre‐existing autoimmunity remains under investigation [181].

In conclusion, enteroviruses remain the most substantiated viral trigger of T1DM. Associations with rotavirus, human herpesviruses, influenza H1N1 and SARS‐CoV‐2 are less consistent and may primarily reflect acceleration of an ongoing autoimmune process rather than disease initiation. Significant challenges remain, including heterogeneity of study populations, diagnostic methods, viral persistence and the paucity of longitudinal paediatric data. Future research should focus on clarifying causal pathways, timing of viral exposure and long‐term β‐cell outcomes in children to inform early intervention and disease prevention strategies.

## 8 Conclusions

Endocrine function plays a pivotal role across all stages of life from fetal development to old age, regulating essential processes such as growth, neurodevelopment, metabolism, gastrointestinal motility, reproductive health, stress response and immune modulation. In particular, the endocrine system contributes significantly to the body's ability to present an effective response to internal and external stressors, including infections.

Despite this central role, the impact of infectious diseases on the endocrine system remains underrecognised and often underestimated in clinical practice.

This review has comprehensively analysed the complex bidirectional interactions between infections and endocrine function, highlighting the importance of considering endocrine complications both during the acute phase of infections and in the post‐infectious period, and the need for long‐term surveillance under some circumstances. Endocrine disruption secondary to infectious diseases can affect quality of life, fertility, metabolic balance and long‐term morbidity and mortality. Therefore, awareness and early identification of endocrine abnormalities should be an integral part of the management of patients with infectious diseases.

Several gaps in knowledge and understanding that would require further research in the field are present. Current evidence is often limited by heterogeneous methodologies, short follow‐up durations and lack of standardised diagnostic criteria. Further translational and clinical research is needed to better define the mechanisms of endocrine involvement, identify ideal biomarkers for early detection, and establish evidence‐based screening and management protocols tailored to specific infections and populations.

Emerging research fields, such as functional genomics, will offer promising opportunities to elucidate the pathophysiological links between infections and endocrine dysfunctions, with the potential to guide patient management strategies in the future. The SARS‐CoV‐2 pandemic has stimulated growing interest in this molecular research area. For instance, Karami et al. [182] integrated transcriptomics with network‐based analyses to highlight dysregulated immune pathways in SARS‐CoV‐2–infected tissues to identify key hub genes with potential therapeutic relevance. Similarly, in the field of endocrinology, high‐dimensional approaches have already provided valuable insights into the relationship between viral infections and autoimmune endocrine disorders, including type 1 diabetes and Hashimoto's thyroiditis [183, 184].

## Author Contributions

**Maria Elisabeth Street:** conceptualization, writing – original draft, methodology, writing – review and editing, supervision. **Esposito Susanna:** conceptualization, writing – review and editing, supervision. **Francesco Capoccia:** investigation, writing – original draft, methodology, writing – review and editing. **Valentina Semeraro:** writing – original draft, methodology, writing – review and editing. **Valentina D'Onghia:** writing – original draft, writing – review and editing, methodology.

## Funding

The authors have nothing to report.

## Conflicts of Interest

The authors declare no conflicts of interest.

## Data Availability

The authors have nothing to report.
